# Validity and reliability of the Swaymeter device for measuring postural sway

**DOI:** 10.1186/1471-2318-11-63

**Published:** 2011-10-20

**Authors:** Daina L Sturnieks, Ria Arnold, Stephen R Lord

**Affiliations:** 1 Neuroscience Research Australia, Barker Street, Randwick, NSW, 2031, Australia

## Abstract

**Background:**

This study aimed to examine: 1) Swaymeter concurrent validity in discriminating between young and older adult populations; 2) Swaymeter convergent validity against a forceplate system; and 3) the immediate test-retest repeatability of postural sway measures obtained from the Swaymeter.

**Methods:**

Twenty-nine older adults aged 71 to 83 years and 11 young adults aged 22 to 47 years had postural sway measured simultaneously with the Swaymeter and a forceplate for three repeat 30 second trials, under four conditions (floor eyes open, floor eyes closed, foam eyes open, foam eyes closed).

**Results:**

Age-related differences in sway parameters across the four conditions were evident using the Swaymeter. Moderate-to-good correlations were found between Swaymeter and forceplate sway measures across conditions (r = 0.560-0.865). Good agreement between the Swaymeter and forceplate were found for anteroposterior and mediolateral sway displacement measures (average offset = 6 mm). Sway path length measures were longer for the forceplate compared to the Swaymeter (average offset = 376 mm), but these data showed good agreement following log-transformation. The Swaymeter was reliable across trials, with intraclass correlation coefficients ranging from 0.654 to 0.944.

**Conclusions:**

The Swaymeter is a reliable tool for assessing postural sway and discriminates between performance of young and older people across multiple sensory conditions.

## Background

The control of standing balance is a task of maintaining the body's centre of mass (COM) within the limits of the base of support, achieved by producing forces on the support surface/s (predominantly under the feet while standing). Excursions of the centre of pressure (COP), the point of application of the ground reaction force, measured by a forceplate has been widely used to represent postural sway, as an index of balance control. However, these measures involve technical devices that can be costly and require processing protocols that can make them unfeasible for many clinics and research facilities.

The need for a simple measure of postural sway exists due to the issue of balance problems and the risk of falls in older people. Increasing age is associated with increases in the magnitude and velocity of postural sway during standing [1,2]. Indeed, postural sway has been shown to be a risk factor for falls in numerous older populations (see [3] for review). One study has shown multiple fallers to have 33-46% greater sway than those who did not experience a fall in the previous 12 months [4]. In a prospective study of 100 adults aged 62-96, the root mean square of the mediolateral COP displacement while standing blindfolded was found to predict those people who fell in a follow-up period of 12 months with a predictive accuracy of 67% [5]. These studies suggest that measuring postural sway, particularly while sensory information is reduced, can provide an indication of an individual's risk of a future fall.

A *low tech *Swaymeter was designed to address the needs of clinicians and researchers with limited resources (e.g. no access to forceplates or motion laboratories). It is a useful field test, as it is compact, lightweight, has short administration and data processing time. Unlike other lightweight and easily applied systems, such as accelerometers and gyroscopes, the Swaymeter involves no electronics or computer processing. Thus, assessment can be conducted in a variety of community settings and health care facilities. Several research groups have found the Swaymeter to be feasible for use in different populations of young and older people [6-8].

The Swaymeter has been used in numerous studies of balance [6-8] as well as retrospective [9,10] and prospective [11,12] investigations of falls risk in older people. Sway path length or sway displacement measures have been found to discriminate between fallers and non-fallers in each of these studies. For example, a prospective study of 341 community-living older women, postural sway assessed with participants standing on a foam mat with eyes open was a significant and independent risk factor for multiple falls [12]. A companion study [20] examined 136 younger women (aged 20-64 years) and reported significant correlations between Swaymeter-recorded postural sway and age, but did not reveal whether postural sway data discriminated between the young and older subgroups. Furthermore, the immediate (intrasession) reliability of the Swaymeter device has not been reported, nor has it been validated against accepted measures of postural sway.

The purpose of this study was to examine: 1) the concurrent validity of the Swaymeter in discriminating between young and older adults; 2) the convergent validity of the Swaymeter against a forceplate system; and 3) the immediate test-retest repeatability of postural sway measures obtained from the Swaymeter. Older adults were hypothesised to have increased postural sway obtained from the Swaymeter, compared to young adults. It was hypothesised that the Swaymeter would show good-to-excellent agreement with forceplate (COP) data, in addition to acceptable reliability across 3 repeated trials.

## Methods

### Participants

Twenty-nine older adults aged 71 to 83 years (mean 78 ± 3) and 11 young adults aged 22 to 47 years (mean age 33 ± 9 years) participated in the study. All were healthy, independent and community-dwelling. Older adults were randomly selected from a database of participants previously involved in a large study of falls risk factors. Young adults were recruited from Institute staff. Exclusion criteria were: neurological; cardiovascular or major musculoskeletal impairments; uncorrected visual or vestibular problems; significant pain or recent injury; poor understanding of the English language; Mini Mental Status Examination [13] score < 24; and unable to walk for 20 m without a walking aid. The study was approved by The University of New South Wales Human Research Ethics Committee and all participants provided informed consent prior to participation.

### Protocol

Assessments of postural sway were performed in bipedal stance, with eyes open and closed, on a firm or compliant (foam) surface, for a total of 4 conditions: floor with eyes open; floor with eyes closed; foam with eyes open; and foam with eyes closed. Conditions were randomly presented and 3 trials of 30 seconds were conducted per condition, for a total 12 trials per participant. Participants were instructed to stand still and without talking, with feet shoulder-width apart and arms crossed over the chest, while looking ahead and slightly down at a blank wall 1.5 m away. Participants were offered a seat and rested for at least 1 minute in between trials. Recordings from Swaymeter and forceplate devices were recorded simultaneously throughout each trial. During compliant surface trials, a medium-density foam rubber mat (15 cm thick, 24 kg/m^3^) was placed directly over the forceplate. The mechanical characteristics of the foam were such that it was compressed to 9 cm when a 50 kg weight was applied equally across its surface.

### Swaymeter

**Figure 1:**
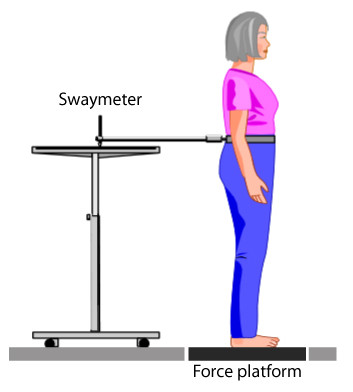
**Pictorial representation of the assessment of postural sway using the Swaymeter and forceplate systems**.

The Swaymeter recorded displacements of the body in the horizontal plane at waist level. The device consisted of an inflexible 40-cm-long rod with a vertically mounted pen at its end. The rod was mounted on a 20 cm wide metal plate which was fitted over the participant's lower back (level of the posterior superior iliac spine) by a firm belt so that the rod extended posteriorly. Fitted firmly, the Swaymeter offers 1 degree of freedom between the belt and pen as it is free to move in the pitch plane. The pen recorded participant's postural sway on a sheet of millimeter graph paper, fastened to the top of an adjustable-height table (Figure 1). The sway path length was manually determined as the number of millimetre squares traversed by the pen [14]. The anteroposterior (AP) and mediolateral (ML) peak-to-peak sway displacements were also calculated from the extremes of sway length in these two planes, as previously described [14].

### Ground Reaction Forces

Ground reaction forces and computed moments were obtained while participants stood on a calibrated 400 × 600 mm Kistler force plate (9286A, Kistler Instrumente AG, Winterthur, Switzerland) embedded flush with the laboratory floor. Forceplate data were acquired using a CODAmotion 64 channel analogue interface (Charnwood Dynamics, Leicestershire, UK), sampling at 1000 Hz. COP co-ordinate calculations were performed by CODAmotion V6.66 software. COP data were smoothed with a low-pass Savitzky-Golay filter, polynomial order 3 and frame length of 41 (MATLAB R2009a, The MathWorks Inc, Massachusetts, USA). COP sway path length was calculated from ML and AP coordinates.

### Statistical analysis

The postural sway variables, calculated from data obtained by the Swaymeter and forceplate, for subsequent analysis were:

1) AP displacement, taken as the maximum position minus the minimum position in the anterioposterior direction;

2) ML displacement, taken as the maximum position minus the minimum position in the mediolateral direction; and

3) Path length, taken as the total sway displacement.

Statistical analyses were performed using SPSS 15.0 for Windows (SPSS Inc, Chicago, USA) with statistical significance set at p < 0.05. Descriptive statistics (means and SD) were calculated for each sway variable, across conditions and age groups (young and old). To examine concurrent validity, between-group differences in log-normalised sway variables were examined using 2-factor repeated measures ANOVAs, with group and condition as factors. Bonferonni post-hoc tests were used to identify significant main effects. To examine Swaymeter convergent validity against the forceplate system, Pearson product moment correlation coefficients were calculated for Swaymeter and COP measures across each condition. In addition, offsets and limits of agreement (95% confidence intervals (CIs)) were calculated according to methods described by Bland and Altman [15]. Bland-Altman plots [15] of AP displacement, ML displacement and sway path length during foam eyes open condition were constructed to illustrate Swaymeter agreement with the forceplate system. The foam eyes open condition was chosen as this is most commonly used for clinical and research purposes [16]. To examine the Swaymeter test-retest reliability across 3 repeated trials, intraclass correlation coefficients (ICC(2,1)), 95% Confidence Intervals (CIs) as well as Standard Error of Means (SEM) were calculated for each condition.

## Results

Data are missing for 2 older adults in the foam eyes closed condition, as they were concerned about their stability and refused to complete the trials. Otherwise, all subjects successfully completed all trials without incident. Table 1 presents participant characteristics and indicates that the study sample comprised relatively healthy groups of young and older people without cognitive impairment.

**Table 1:** Anthropometric, fall risk, fall history, health and medical characteristics of young and older participants.

Mean (SD)	Old (n = 29)	Young (n = 11)
Height (cm)	164.4 (9.9)	170.9 (6.7)
Weight (Kg)	72.5 (9.9)	66.3 (14.9)
Physiological Profile Assessment (PPA) falls risk score	0.60 (1.06)	-
Short-form 36 general health score (range 0 - 100)	70.4 (16.9)	-
Mini Mental Status Examination score (range 0 - 30)	27.8 (1.7)	-

**Number (%)**		

Female gender	14 (48.3)	6 (54.5)
One or more falls in previous year	7 (24.1)	-
Two or more medical conditions*	24 (82.8)	-
Four or more medications	18 (62.1)	-

*Medical conditions surveyed were; peripheral vascular disease, diabetes, stroke, trans-ischemic attack, heart attack, angina, high blood pressure, heart/blood vessel problems, and arthritis.

Descriptive statistics for sway displacement and path length from Swaymeter recordings in the young and older participants are presented in Table 2. Significant group differences existed in Swaymeter variables, with older adults having greater AP displacement, (F_1,473 _= 23.84, p < 0.0001), ML displacement (F_1,473 _= 50.94, p < 0.0001) and path length (F_1,473 _= 54.29, p < 0.0001) than young. Furthermore, there was a significant main effect for condition (F_3,473 _= 53.47, p < 0.0001), with Bonferroni post-hoc tests showing significant differences between all conditions (p < 0.024). The group*condition main effect was not significant (F_3,473 _= 0.13, p = 0.943).

**Table 2:** Mean (SD) sway measures (mm) for young and older participants, as determined from Swaymeter recordings.

	Young (n = 11)						Old (n = 29)				
	**Floor**			**Foam**			**Floor**			**Foam**	
	**Eyes open**	**Eyes closed**		**Eyes open**	**Eyes closed**		**Eyes open**	**Eyes closed**		**Eyes open**	**Eyes closed**

AP displacement	15.98 (5.35)	19.17 (8.71)		22.86 (10.11)	33.48 (13.45)		20.36^+^ (6.99)	26.09* (10.79)		25.90 (7.86)	46.80* (19.51)

ML displacement	12.61 (5.24)	15.64 (8.54)		19.83 (6.31)	29.17 (10.64)		23.08* (11.50)	27.07* (15.43)		32.93* (17.79)	52.02* (23.42)

Path length	59.33 (25.98)	83.97 (34.08)		108.27 (46.72)	195.03 (51.81)		106.00* (44.96)	135.08* (62.91)		186.54* (74.25)	359.36* (169.72)

*significantly different to Young (p < 0.01)

^+^significantly different to Young (p < 0.05)

### Swaymeter Convergent Validity

Table 3 presents Pearson correlation coefficients between Swaymeter and forceplate (COP) data. The Swaymeter measures were moderately to strongly associated with COP measures, with correlation coefficients between 0.560 and 0.865. In particular, the Swaymeter had excellent correlation with COP for the AP displacement measure, with correlation coefficients greater than 0.74 across all conditions.

**Table 3:** Pearson correlation coefficients (r), followed by Bland-Altman offsets (mm) and limits of agreement (95%CIs) for sway measures, as determined from Swaymeter recordings, against those taken from forceplate (COP) recordings.

	Floor		Foam	
	Eyes open	Eyes closed	Eyes open	Eyes closed
AP displacement *Offset (95%CI)*	r = 0.743 *4.5(-6.8-15.7)*	r = 0.845 *6.8(-3.2-16.7)*	r = 0.820 *9.5(-4.9-23.8)*	r = 0.865 *15.6(-5.2-36.4)*

ML displacement *Offset (95%CI)*	r = 0.692 *-4.1(-19.5-11.3)*	r = 0.752 *-5.4(-25.0-14.2)*	r = 0.733 *0.1(-19.0-19.3)*	r = 0.807 *-2.7(-30.1-24.7)*

Path length *Offset (95%CI)*	r = 0.667 *231.0(68.6-393.4)*	r = 0.754 *275.5(-58.1-609.1)*	r = 0.560 *409.2(55.1-763.3)*	r = 0.858 *590.0(-363.9-1543.9)*

**Figure 2:**
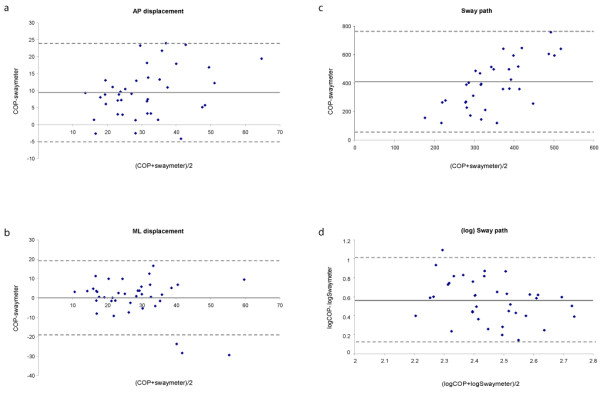
**Bland-Altman plots of: a) AP displacement (mm); b) ML displacement (mm); c) sway path length (mm); and d) log sway path length, for Swaymeter versus forceplate (COP) comparisons during the foam eyes open condition**. The dashed grey lines represent the limits of agreement (95%CI) from the mean difference, represented by the solid grey line.

Table 3 also presents offsets and limits of agreement between Swaymeter and forceplate (COP) measures. Since Bland-Altman plots were similar across conditions, representative plots are presented in Figure 2. These plots indicate good agreement between Swaymeter and COP data for AP and ML displacement (Figure 2a, b), since few datapoints fall outside of the limits of agreement (95% CI). The mean Swaymeter-COP offset for AP displacement indicates increased magnitude recorded from the forceplate system, which was similar across the range of data. Minimal Swaymeter-COP offsets were seen for ML displacement. The sway path length was considerably larger for COP compared to Swaymeter data, with the difference increasing with the magnitude of sway (Figure 2c). Following log-transformation of sway path datasets, the relationship between Swaymeter and faceplate sway path was consistent across the range of data (Figure 2d).

### Repeatability

ICCs (95% CIs) and SEMs for Swaymeter data recorded during 3 repeated trials are presented in Table 4. Swaymeter measures showed good-to-excellent repeatability across conditions, with ICCs ranging from 0.654 to 0.944. Path length measures showed particularly high repeatability, with all ICCs in excess of 0.83.

**Table 4:** Intraclass correlation coefficients (95% CIs) and Standard Error of Means (SEM) for sway measures across four conditions, as determined from Swaymeter recordings.

	Floor		Foam	
	Eyes Open	Eyes Closed	Eyes Open	Eyes Closed
AP displacement	.654 (.414-.806) SEM = .014	.774 (.618-.873) SEM = .017	.666 (.438-.812) SEM = .013	.777 (.616-.877) SEM = .017

ML displacement	.763 (.597-.867) SEM = .022	.821 (.697-.899) SEM = .024	.792 (.650-.883) SEM = .020	.823 (.697-.902) SEM = .021

Path length	.834 (.720-.906) SEM = .020	.944 (.906-.969) SEM = .019	.894 (.816-.941) SEM = .019	.933 (.885-.963) SEM = .021

## Discussion

Control of the whole body COM is the primary goal of the balance system. The Swaymeter provides an indirect measure of COM movement as it is fixed at approximately the level of the COM (pelvis) and records motion of the body in 2D while standing. In order to stabilise the COM, an individual produces forces on the support surface/s (predominantly under the feet while standing). Due to the relative ease of forceplate recordings, the COP has commonly been used in previous studies of standing balance control as an indicator of balance stability. However, while COM and COP measures are related, they are not synonymous [17]. The COP-COM position separation varies across individuals and conditions. Despite this, the Swaymeter showed good agreement with forceplate COP measures for AP and ML displacement (average offset = 6 mm) and moderate-excellent correlations for AP (r > 0.743) and ML displacement (r > 0.692).

While moderate correlations (r > 0.560) were found for Swaymeter versus forceplate sway path length, Bland-Altman plots revealed measures were consistently longer for the forceplate compared to the Swaymeter (average offset = 376 mm). Since correlations tend to increase when the data are more widely spread, the Bland-Altman statistics provide more meaningful detail regarding measurement agreement. The Bland-Altman results show that the magnitude of difference between Swaymeter and forceplate measures of sway path increased with the magnitude of sway. This result may be due to the reduced precision of the Swaymeter (1 mm), the dampening of body motion through the Swaymeter and pen-paper interface friction, and the higher frequency motions recorded by the forceplate system. Increased filtering of COP data might improve the agreement between these measures. However, these results suggest that postural sway path length cannot be directly compared between Swaymeter and forceplate recordings. Subsequent analyses on log-transformed sway path datasets showed good agreement across the range, indicating that data from these sources may be compared following log-transformation. However, it should be noted that log transformations of Swaymeter data are not required in clinical settings, as raw scores can be contrasted to population norms to provide an indication of an individual's postural stability [16].

Immediate test-retest repeatability was found to be good for all Swaymeter measures across the 4 conditions. The correlation coefficients across trials were excellent for sway path length (ICC > 0.834) and ML displacement (ICC > 0.763), and good for AP displacement (ICC > 0.654). Data from all participants were included in these analyses to cover a fuller range (spread) of data points, as the issue of interest was the device, not the participant group. However, similar associations were evident when the analyses were restricted to the older group alone (data not shown). The superior Swaymeter ICCs in the ML plane, relative to AP, are in agreement with those previously reported for within-day COP velocity ICCs [18,19]. Within session reliability is largely related to the random variability of the measurement, as opposed to intersession reliability which may incorporate changes in postural stability over a longer period of time and errors associated with reapplication of setup protocols. Lin and colleagues [18] also found within-day reliability to be better than between-day reliability for both young and older adult groups. Similarly, we found higher reliability coefficients for immediate measures compared with those previously measured for repeats over a longer period [4], which suggests that an individual's postural sway may change over days or weeks.

Sway variables measured with the Swaymeter significantly differed between young and older participants, in line with previous findings [20]. These differences provide evidence of Swaymeter concurrent validity for examining age-related differences in postural sway. One quarter of our older adult group reported a fall in the previous year, suggesting that we recruited a representative sample of healthy community-dwelling older adults living in the Australian state of New South Wales [21]. The between-group differences reported here are, therefore, indicative of normal age-related declines in postural sway in healthy community-dwelling older people.

Maki and colleagues [5] have suggested that a quick, simple, and safe measure of postural sway provides a better prediction of future falling risk in older people compared with measures derived using more complicated and expensive moving platform protocols that induce balance responses. Piirtola and Era [3] reviewed nine prospective studies of forceplate sway measures and found only five to be significantly associated with falls outcomes in older people. In contrast, Swaymeter measures have consistently shown significant associations with future falls across multiple populations of older people (ie people living in both the community and residential care) [11,12,22]. The consistent findings most likely relate to portability and feasibility factors, which have allowed assessment of large sample sizes (with resultant increased statistical power) and assessment of older people across a broad stability spectrum (with resultant high inter-participant sway variability). It is also possible that apparent limitations of the Swaymeter, including the cross-talk between ML and yaw motion (twisting) resulting in amplified Swaymeter-recorded ML displacement and sway path length and/or the dampening of pelvis motion through the device and pen-paper interface friction, provide measures that are more predictive of future falls than forceplate COP measures. For example, future fallers may have more yaw motion while standing, which is detected by larger ML displacement from Swaymeter recordings but not seen with COP measures. It should be noted that the Swaymeter is subject to operator error, as sway variables are counted and computed manually.

## Conclusions

The Swaymeter enables measurement of postural sway that is simple and less expensive than methods employing forceplate or motion capture systems. This study has found the Swaymeter to be a reliable measure of postural sway that discriminates between young and older people across multiple sensory conditions. Sway displacement measures from the Swaymeter agreed well with forceplate measures, while sway path measures showed an offset that increased with the magnitude of sway, which can be overcome by correcting the skewed data via log-transformation.

## List of abbreviations

AP: Anteroposterior; CI: Confidence intervals; COM: Centre of mass; COP: Centre of pressure; ICC: Intraclass correlation coefficients; ML: Mediolateral; SEM: Standard error of the mean; SD: Standard deviation.

## Competing interests

The Swaymeter is commercially available from Neuroscience Research Australia. The authors declare that they have no other competing interests

## Authors' contributions

DS conceived the study, participated in its design and coordination, collected and processed the data, helped with statistical analyses and drafted the manuscript. RA reviewed literature, helped with statistical analyses and drafted the manuscript. SL participated in the study design and coordination, helped with statistical analyses and drafted the manuscript. All authors have read and approve the final manuscript.

## Pre-publication history

The pre-publication history for this paper can be accessed here:

http://www.biomedcentral.com/1471-2318/11/63/prepub
